# An exaggerated blood pressure response to exercise is associated with attenuated exercise‐induced acute cognitive improvement: A pilot study

**DOI:** 10.14814/phy2.70328

**Published:** 2025-05-22

**Authors:** Narumi Kunimatsu, Hayato Tsukamoto, Takuro Washio, Shotaro Saito, Marino Karaki, Hervé Normand, Shigehiko Ogoh

**Affiliations:** ^1^ Department of Biomedical Engineering Toyo University Asaka Japan; ^2^ Faculty of Sport Sciences, Waseda University Tokorozawa Japan; ^3^ Institute for Exercise and Environmental Medicine at Texas Health Presbyterian Hospital Dallas Dallas Texas USA; ^4^ University of Texas Southwestern Medical Center Dallas Texas USA; ^5^ School of Health and Sport Sciences, Chukyo University Toyota Japan; ^6^ Université de CAEN Normandie Caen France

**Keywords:** blood pressure, cerebral blood flow, cognitive function, static exercise

## Abstract

Some individuals, despite having normal resting blood pressure, exhibit an exaggerated blood pressure response during exercise, indicating a potential risk for future hypertension. This study aimed to investigate how different individual blood pressure responses to exercise affect cerebral circulation and exercise‐induced acute cognitive changes in young, healthy individuals. To eliminate the influence of aging and disease, thirty young, healthy individuals (aged 21 ± 1 years) participated in this study. They performed an interval static handgrip exercise protocol, during which arterial blood pressure (ABP), cognitive function, and middle cerebral artery blood velocity (MCA V), as an index of cerebral blood flow, were measured. Cognitive function was assessed using the Go/No‐go test before exercise and 3 min after exercise completion. Individual changes in systolic blood pressure (SBP) were significantly and linearly related to the decrease in reaction time during a cognitive task, indicating cognitive improvement following exercise (*p* < 0.01). Importantly, in the top 10 subjects with the highest SBP responses (*n* = 10, + 38 ± 8 mm Hg), this cognitive improvement was not statistically significant (*p* = 0.32). These findings suggest that an exaggerated ABP response to exercise may compromise acute cognitive enhancements induced by exercise in young individuals.

## 1 INTRODUCTION

Exercise has been utilized for both the treatment and prevention of various chronic conditions, including heart disease, pulmonary disease, diabetes, obesity, and dementia. In 2007, the American College of Sports Medicine (ACSM) introduced the concept of “exercise is medicine” (Thompson et al., 2020). Exercise has been shown to improve cognitive function in young healthy individuals (Ogoh et al., 2014; Washio et al., 2021). Identifying the mechanisms underlying this effect is crucial for developing effective exercise protocols that enhance acutely cognitive function and potentially prevent dementia. In addition to exercise intensity and mode (Saito et al., 2021), previous studies suggest exercise‐induced changes in cerebral blood flow (CBF), along with the peripheral vascular response, specifically the arterial blood pressure (ABP) response to exercise, could significantly influence acute improvements in cognitive function (Muller et al., 2007; Ogoh, 2017). However, the underlying physiological mechanisms by which exercise improves cognitive function remain unclear.

Interestingly, a previous study (Teixeira et al., 2019) has shown that patients with hypertension who exhibit an exaggerated ABP response to exercise experience altered acute improvement in cognitive function following 12 weeks of exercise. Such exaggerated ABP responses may lead to a more significant increase in CBF, resulting in a greater shear rate that activates endothelin function (Iwamoto et al., 2020; Sakamoto et al., 2024), potentially enhancing cognitive function acutely (Freeberg et al., 2023; Katusic et al., 2023; Trigiani et al., 2022). In contrast, exaggerated ABP responses may damage blood vessels and increase the risk of cerebral and cardiovascular disease (Mundal et al., 1994; Schultz et al., 2015). Moreover, these responses have been associated with an elevated risk of future hypertension, even in young healthy individuals with normal resting ABP (Singh et al., 1999). These findings suggest that an exaggerated ABP response may diminish the positive acute effects of exercise in even young healthy individuals. It is noteworthy that variation in ABP response to exercise has been observed not only in older populations with cardiovascular disease but also in healthy young individuals (Fisher et al., 2008; Mundal et al., 1994; Schultz et al., 2015; Washio et al., 2021). Some individuals, despite having normal resting blood pressure, exhibit an exaggerated blood pressure response during exercise. Reports indicate that 9%–26% of young, healthy individuals show an exaggerated ABP response to exercise (Lauer et al., 1992). Interestingly, our previous study has shown a negative relationship between an individual's ABP response to acute handgrip (HG) exercise and acute cognitive improvement, suggesting that individuals with a low ABP response to exercise experience enhanced cognitive function acutely, while this effect may not be observed in those with a higher ABP response to exercise (Washio et al., 2021). Nonetheless, this finding remains controversial due to limitations in this study, such as the small sample size (Washio & Ogoh, 2023). However, the full impact of these variations in ABP response on cerebral circulation remains unclear. Additionally, it is uncertain whether individual differences in ABP response in healthy young individuals with normal resting BP, particularly an exaggerated ABP response, may influence exercise‐induced acute cognitive improvement.

In the present study, we hypothesized that an exaggerated ABP response to exercise would lead to a more significant increase in CBF in young individuals but would not necessarily enhance cognitive function. We also aimed to determine whether different individual ABP responses modify the positive acute effect of exercise on cognitive function. To test this hypothesis, we investigated the acute effects of individual variations in ABP, particularly an exaggerated ABP response, on cerebral circulation and cognitive function in young healthy individuals. This finding is very important for determining adequate exercise rehabilitation, etc. Some individuals may believe that exercise acutely improves cognitive function but may not actually experience this benefit.

## 2 METHODS

The study was approved by Toyo University's Institutional Review Board (TU‐2018‐023, TU‐2021‐028), and all participants provided written informed consent in accordance with the Declaration of Helsinki. Thirty young, healthy volunteers (24 men and 6 women; age 21 ± 1 years) participated. They were nonsmokers, right‐handed, free from cerebrovascular or cardiovascular disease, and not taking any medications. Participants were recreationally active but not competitive athletes. Before the experiment, they abstained from caffeine for 12 h, refrained from strenuous exercise, and avoided alcohol for 24 h.

**FIGURE 1:**
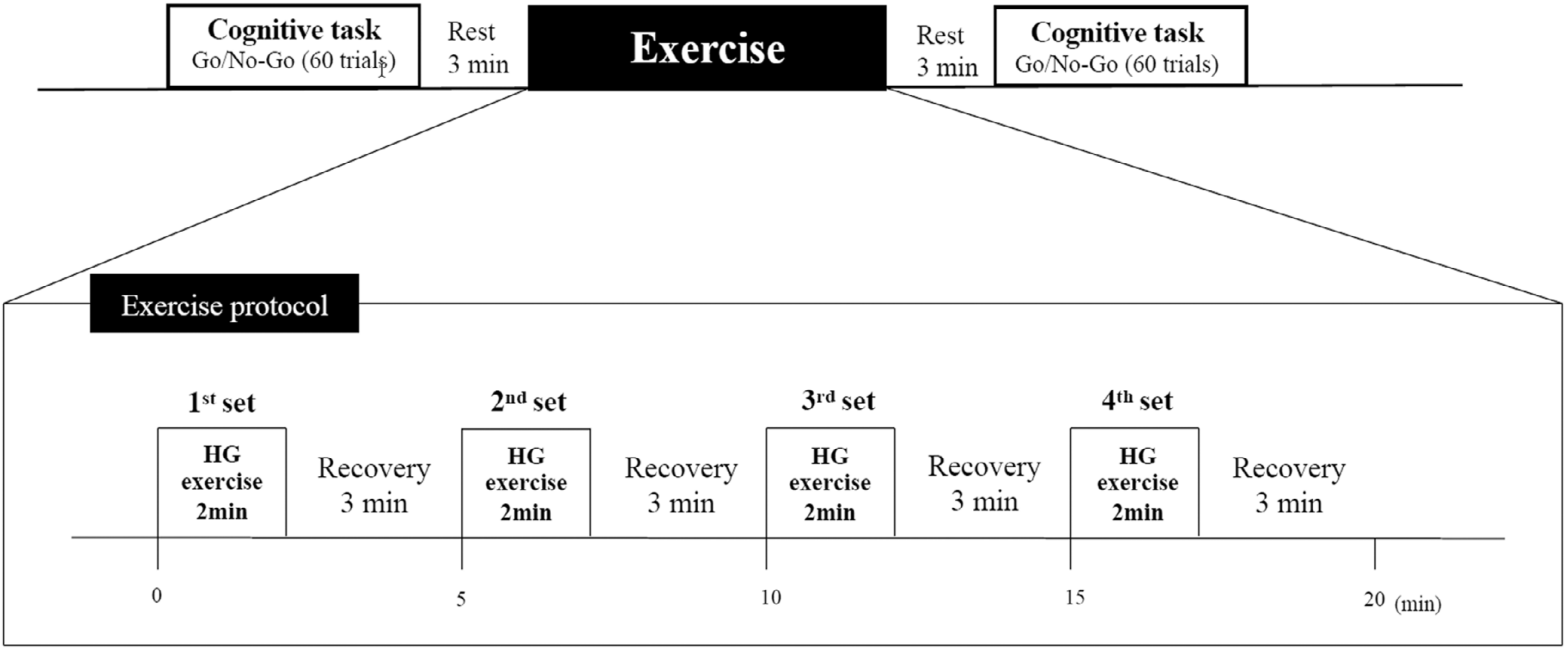
Overview of the experimental protocol.

Participants performed 2–3 maximal voluntary static handgrip contractions with their nondominant hand (left hand) to determine exercise intensity. They also completed at least 30 trials of the Go/No‐go task to stabilize reaction times. Following this, they underwent an interval handgrip (IHG) exercise protocol consisting of four trials of 2 min each at 25% of maximal voluntary contraction (MVC), with 3‐min rests between trials. Cognitive tasks were performed before the start of the exercise protocol and 3 min after its completion (Figure 1). In the additional study, we have already confirmed that there is no time effect on cognitive function (*n* = 13, Baseline vs. 25 min rest which is matched with cognition measurement in this exercise protocol, RT 373 ± 40 vs. 377 ± 40 ms, *p* = 0.544 and performance accuracy 99 ± 1 vs. 99 ± 1%, *p* = 0.581); therefore, we did not include a control condition to reduce participant stress and shorten the protocol. This IHG exercise protocol has been indicated as the method for reducing resting ABP in patients with hypertension (Yamagata & Sako, 2020).

Heart rate (HR) was monitored using a lead II electrocardiogram (bedside monitor, BMS‐3400; Nihon Kohden, Tokyo, Japan). ABP was continuously monitored using finger photoplethysmography (Finapres Medical Systems, Amsterdam, the Netherlands). The partial pressure of the end‐tidal carbon dioxide (P_ET_CO_2_) was measured with a capnometer (OLG‐3800, Nihon Kohden). The middle cerebral artery mean blood velocity (MCA Vm) on the right side was determined using transcranial Doppler ultrasonography (TCD, DWL Doppler Box‐X; Compumedics, Singen Germany). The TCD probe was securely affixed to the right temple using an elastic headband (Elastic headband, DWL). All data were sampled continuously at 1 kHz using an analog‐to‐digital converter (PL3516 PowerLab 16/35, ADInstruments) and stored on a laboratory computer for subsequent offline analysis. Cognitive function was assessed using the Go/No‐go task, measuring reaction time (RT) and accuracy before and after the IHG exercise protocol. The Go/No‐go task was programmed using Presentation software (Presentation ver. 19; Neuro Behavioral System, Berkeley, CA, USA). Cerebrovascular and cardiovascular variables were averaged using 30‐s data points before (Baseline) and at the last 30 s of the fourth trial of the IHG exercise protocol.

Data are presented as mean ± standard deviation (SD) when normal data distribution was confirmed using the Shapiro–Wilk tests. For nonnormally distributed data, results are expressed as median [IQR]. Statistical analysis was performed using a paired two‐tailed *t*‐test for normally distributed data and the Wilcoxon signed‐rank test for nonnormally distributed data. Statistical significance was set at *p* < 0.05. Effect sizes were calculated using Cohen's *d* for the paired *t*‐test and *r* for the Wilcoxon signed‐rank test. Pearson correlation coefficient was used to assess the relationship between the changes in SBP and mean MCA V or RT from baseline to EX values.

## 3 RESULTS

**FIGURE 2:**
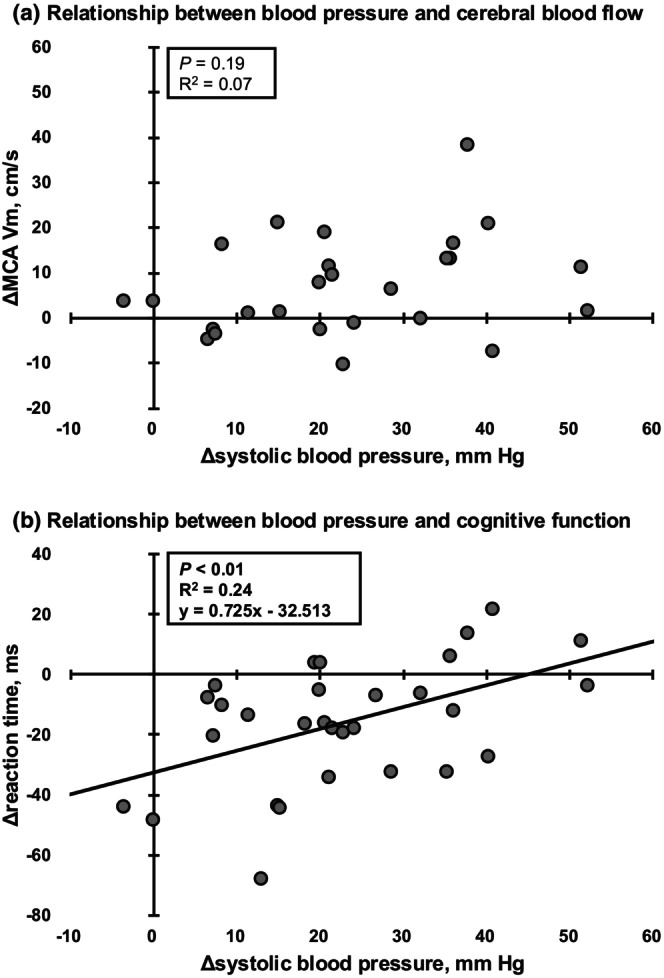
The relationships between changes in systolic blood pressure and middle cerebral artery mean blood velocity (MCA Vm) (a), changes in systolic blood pressure and reaction time of Go/No‐go test (b) in response to handgrip exercise. Four subjects were excluded from the analysis due to excessive noise in the MCA V signals.

The IHG exercise increased HR (*p* < 0.01, *r* = 0.84), SBP (*p* < 0.01, *d* = 1.22), MAP (*p* < 0.01, *d* = 1.60), DBP (*p* < 0.01, *d* = 1.65), and MCA Vm (*p* < 0.01, *d* = 0.47), but not P_ET_CO_2_ (*p* = 0.28, *r* = 0.20, Table 1). Notably, change in SBP during the handgrip exercise protocol across 30 subjects followed a normal distribution (*p* = 0.70) but showed large variation (−4 to 52 mm Hg). The RT of the Go/No‐go task was reduced after the IHG exercise protocol compared to baseline (*p* < 0.01, *d* = 0.43), indicating improved cognitive function, while task accuracy remained unchanged (*p* = 0.48, *r* = 0.01). Regression analysis was conducted to examine the relationship between changes in SBP and RT in the Go/No‐go task or mean MCA V. The results showed that the individual change in SBP was significantly related to the decrease in RT during the cognitive task, indicating cognitive improvement following exercise (*p* < 0.01, *R* ^2^ = 0.24, Figure 2). However, no significant relationship was observed between change in SBP and mean MCA V (*p* = 0.19, *R* ^2^ = 0.07). Importantly, the average RT for all subjects deceased (*p* < 0.01, *d* = 0.43, Table 1). However, in the top 10 individuals with the highest SBP responses (*n* = 10, + 38 ± 8 mm Hg), this cognitive improvement was not statistically significant (*p* = 0.32, *d* = 0.19). In contrast, significant cognitive improvement was observed in the low and middle SBP response groups (low, *n* = 10, + 8 ± 6 mm Hg, *p* < 0.01, *d* = 0.33: middle, *n* = 10, + 21 ± 3 mm Hg, *p* = 0.04, *d* = 0.74).

**TABLE 1:** Average of hemodynamic changes and cognitive function in response to isometric handgrip.

	Baseline	Exercise	*p* Values	Effect size
HR, bpm	68 [62–77]	81 [75–88]	<0.01	*r* = 0.84
SBP, mm Hg	124 ± 14	146 ± 22	<0.01	*d* = 1.22
MAP, mm Hg	91 ± 10	112 ± 15	<0.01	*d* = 1.60
DBP, mm Hg	73 ± 8	89 ± 11	<0.01	*d* = 1.65
MCA Vm, cm/s	61 ± 13	68 ± 17	<0.01	*d* = 0.47
P_ET_CO_2_, mm Hg	35 [33–38]	36 [32–38]	0.28	*r* = 0.20
Reaction time, ms	376 ± 38	360 ± 36	<0.01	*d* = 0.43
Performance accuracy, %	100 [98–100]	100 [98–100]	0.48	*r* = 0.01

*Note*: Data are mean ± standard deviation (SD) or median [interquartile range (IQR)]. Average reaction time and performance accuracy indicated the scores of Go/No‐go test. Four subjects were excluded from the analysis due to excessive noise in the MCA V signal (i.e., *n* = 26).

Abbreviations: DBP, diastolic blood pressure; HR, heart rate; MAP, mean arterial pressure; MCA Vm, middle cerebral artery mean blood velocity; P_ET_CO_2_, partial pressure of the end‐tidal carbon dioxide; SBP, systolic blood pressure.

## 4 DISCUSSION

The present study found significant individual variation in ABP response to exercise among young, healthy individuals. An exaggerated ABP response has been linked to a higher risk of developing hypertension, even in individuals with normal resting ABP (Bond et al., 2012; Singh et al., 1999; Smith et al., 2006). As a result, while most young, healthy individuals experienced acute cognitive benefits from interval HG exercise, those with a higher increase in SBP during exercise showed lower acute cognitive improvement despite having normal resting blood pressure (Figure 2). Specifically, individuals with a higher SBP response to exercise exhibited a significant lack of exercise‐induced cognitive improvement. This suggests that an inadequate cardiovascular response to exercise may be associated with reduced acute effectiveness of exercise on cognitive function, even in healthy individuals.

As with previous studies (Matthews et al., 1998; Miyai et al., 2002), the present study found significant individual variation in ABP responses to exercise, with some individuals showing exaggerated ABP response despite normal resting ABP. Such exaggerated responses may indicate an increased future risk of developing hypertension (Matthews et al., 1998). This response could serve as an early warning sign of abnormal blood pressure regulation, often undetected by routine clinical measurements. The exact mechanism behind this exaggerated response in individuals with normal resting ABP is not fully understood. Factors such as family history of hypertension (Greaney et al., 2015), left ventricular mass (Oh et al., 2018), arterial stiffness (Wuttichaipradit et al., 2024), metabolic syndrome (Gaudreault et al., 2013), and exercise capacity (Kokkinos et al., 2002) have been linked to exaggerated ABP responses to exercise. Given that arterial stiffness has been shown to influence cognitive dysfunction (Mason et al., 2022; Muela et al., 2018), it may contribute to the diminished acute cognitive benefits observed in individuals with exaggerated ABP responses during HG exercise, despite normal resting blood pressure.

A possible mechanism behind exercise‐induced acute cognitive improvements is thought to be elevated CBF. While increased CBF has been linked to acute enhanced cognitive function (Tari et al., 2020), its impact remains controversial (Ogoh et al., 2014). In the present study, the exercise‐induced increase in MCA Vm, an index of CBF, was not significantly associated with an increase in SBP responses (*p* = 0.19, *R* ^2^ = 0.07 Figure 2). This MCA Vm response, which is associated with ABP, is likely due to attenuated cerebral autoregulation. However, since HG exercise does not impair dynamic cerebral autoregulation (Ogoh et al., 2010) and results of the present study showed that the MCA Vm response was not associated with the SBP response, the MCA Vm response to changes in ABP during HG exercise remains controversial. For instance, aging increases the ABP response to HG exercise, but instead of increasing, CBF decreases (Fisher et al., 2008).

Despite exercise‐induced increases in CBF, cognitive function did not improve acutely in those with higher BP responses, particularly in the top ten subjects with the highest BP response to exercise (*p* = 0.32, *d* = 0.19). This suggests that an increase in CBF with a higher ABP response may not necessarily enhance cognitive function acutely during HG exercise. Our recent study (Ogoh et al., 2024) investigated the effect of experimentally manipulated changes in CBF on brain neural activity related to motor execution and inhibitory processing using electroencephalographic event‐related potentials (EEG‐ERPs). The study demonstrated that changes in CBF did not impact either executive or inhibitory functions. These findings, along with our results, suggest that exercise‐induced acute cognitive improvement is not solely due to changes in CBF. Interestingly, cognitive performance acutely improved during prolonged heavy exercise despite the decrease in CBF (Ogoh et al., 2014). These findings suggest that acute exercise‐induced cognitive improvement may not have the same narrative as the chronic effects of exercise in terms of the cerebrovascular system. This finding implies that another factor, modified by exercise and encompassing both acute and chronic influences, rather than an increase in CBF, is associated with exercise‐induced improvements in cognitive performance (Hashimoto et al., 2021).

Our previous studies (Washio et al., 2021) showed a negative linear correlation between exercise‐induced acute cognitive improvements and elevated ABP responses. In the present study, our current data confirmed the linear relationship between cognitive improvement and elevated SBP response to exercise, using a larger sample size compared to these previous studies (Washio et al., 2021). In addition, this linear equation derived from our result (RT = SBP*0.725–32.513) suggests that an SBP increase exceeding approximately 45 mm Hg predicts no reduction in RT during the cognitive task. Thus, individuals with a higher SBP response are unable to achieve exercise‐induced cognitive improvement. Indeed, among the top 10 individuals with the highest SBP response (*n* = 10, + 38 ± 8 mm Hg), cognitive improvement was not statistically significant (*p* = 0.32, *d* = 0.19). In contrast, significant cognitive improvements were observed in the low and middle SBP response groups in this study (low, *n* = 10, + 8 ± 6 mm Hg, *p* < 0.01, *d* = 0.33: middle, *n* = 10, + 21 ± 3 mm Hg, *p* = 0.04, *d* = 0.74). These findings indicate that only an exaggerated ABP response was linked to reduced more acute cognitive benefits from exercise, indicating that the relationship between acute cognitive improvements and elevated SBP may be more complex than a simple negative correlation. The study suggests that conditions like exaggerated ABP response may be related to reduced acute cognitive benefits of exercise.

Exercise intensity affects the acute impact of exercise on cognitive function, with moderate exercise potentially being the most cognitive function most efficiently improvement (Kamijo et al., 2004). Meta‐analysis supports this effect of exercise intensity (Zhang et al., 2023), highlighting the need for precise information on appropriate exercise intensity and mode to maximize acute cognitive benefits. Importantly, tailoring exercise intensity based on an individual's ABP response may further enhance these acute benefits. Crucially, individuals with exaggerated exercise‐induced blood pressure may be unaware of their condition, potentially continuing to exercise with the intention of preventing cognitive decline without experiencing the anticipated benefits. Therefore, monitoring blood pressure during exercise, alongside resting measurements, may be crucial for identifying such issues. HG exercises and monitoring ABP responses may be useful tools for exercise prescriptions. For individuals with exaggerated ABP responses (e.g., older adults, hypertensive patients, and young individuals with high ABP responses), it may be vital to adjust exercise workload, mode, or duration is vital to prevent diminished acute cognitive benefits.

This study has some limitations that should be considered. First, a potential limitation of the transcranial Doppler ultrasonography measurement should be noted. Vasoconstriction of the insolated vessel increases MCA V at any given volume of flow. However, in humans, the MCA diameter appears to remain relatively constant under a variety of conditions (Schreiber et al., 2000; Serrador et al., 2000; Zotcheva et al., 2022). Thus, we would argue that the beat‐to‐beat changes in MCA V reflect changes in flow at rest (Ogoh et al., 2005; Zotcheva et al., 2022). Second, the unequal gender distribution across groups. Sex differences and hormonal status might have influenced our results since both men and women were included in this study, and women's menstrual cycles were not controlled. Therefore, hormonal variations could influence the findings, and further research is needed to address this issue. Finally, this study assessed cognitive function solely based on RT using the Go/No‐go task, which limits the generalizability of these findings to other cognitive tests and domains.

## FUNDING INFORMATION

This work was supported by JSPS KAKENHI (no. JP23K24727) from the Japanese Ministry of Education, Culture, Sports, Science, and Technology.

## CONFLICT OF INTEREST STATEMENT

The author(s) declared no potential conflicts of interest with respect to the research, authorship, and/or publication of this article.

## Data Availability

The data that support the findings of this study are available on request from the corresponding author.
